# Supplementary material for: Exposure Assessment of Environmental Tobacco Aerosol from Heated Tobacco Products: Nicotine and PM Exposures under Two Limited Conditions
Source: Int J Environ Res Public Health. 2020 Nov 18;17(22):8536. doi: 10.3390/ijerph17228536 (PMC7698763; doi:10.3390/ijerph17228536)
Supplement: Supplementary file 1 [file ijerph-17-08536-s001.zip › ijerph-961167-supplementary.docx]

Supplementary Materials

Exposure assessment of environmental tobacco aerosol from heated tobacco products: nicotine and PM exposures under two limited conditions

Tomoyasu Hirano^1, †,*^, Tokuaki Shobayashi^1^, Teiji Takei^1^ and Fumihiko Wakao^2^

^1^ Health Service Division, Health Service Bureau, Ministry of Health, Labour and Welfare, Government of Japan, Tokyo 100-8916, Japan; [shobayashi-tokuaki@mhlw.go.jp](mailto:shobayashi-tokuaki@mhlw.go.jp) (T.S.); [takei-teiji@mhlw.go.jp](mailto:takei-teiji@mhlw.go.jp) (T.T.)

^2^ Center for Cancer Control and Information Services, National Cancer Center, Japan, Tokyo 104-0045, Japan; [fwakao@ncc.go.jp](mailto:fwakao@ncc.go.jp)

^†^ Current address: Division of Tobacco Policy Research, National Cancer Center, Tokyo 104-0045, Japan

***** Correspondence: [tohirano@ncc.go.jp](mailto:tohirano@ncc.go.jp)

Supporting Information 1


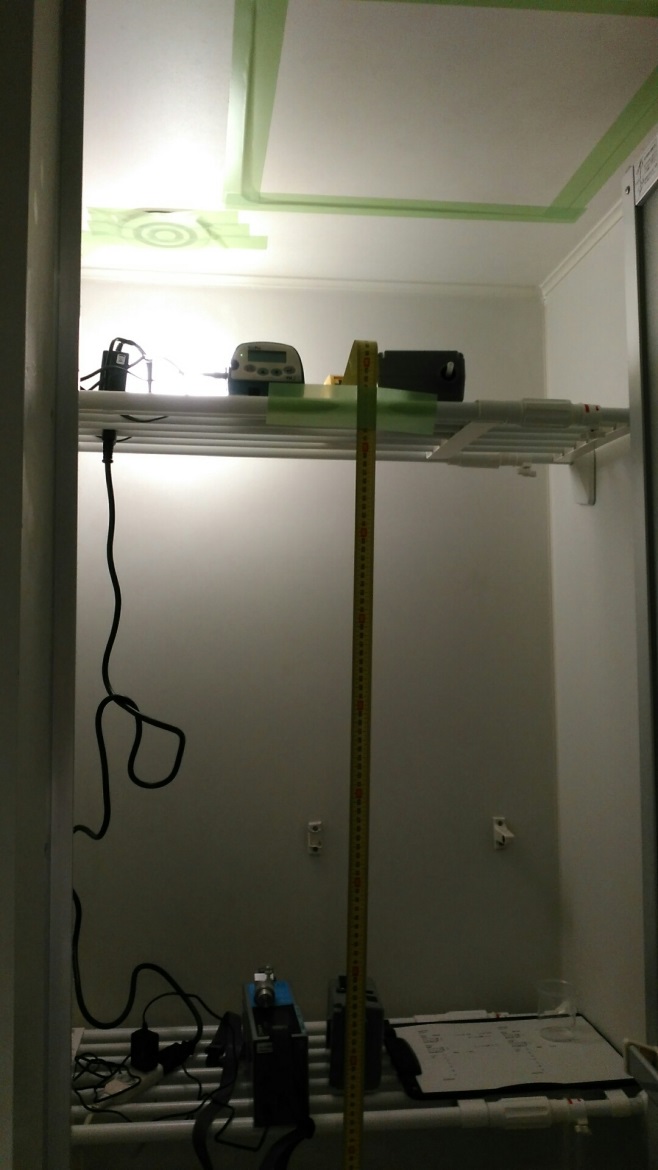


**Figure S1.** Photo of the tested shower cubicle.

Length, width, and height were 0.80 m, 0.80 m, and 2.24 m, respectively. Ventilation was prevented by covering the ventilation fan and drainage with vinyl tape. Strut-pole shelves were set at heights of 1.8 m and 1 m, on which nicotine collecting cartridges and PM2.5 measuring instruments were placed.

Supporting Information 2


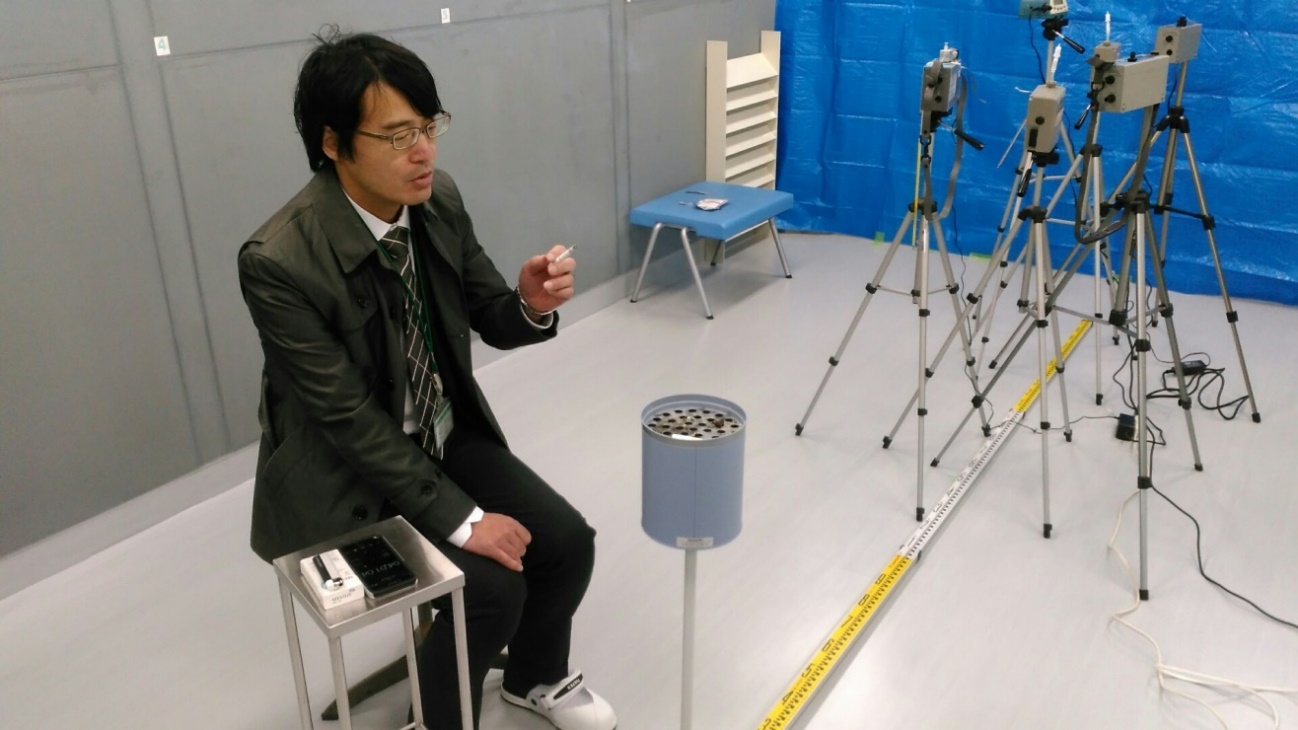


**Figure S2.** Photo of the test setup.

The room was 25 m^2^. Vinyl sheeting covered the walls. The nicotine collecting cartridge carrier and PM_2.5_ measuring equipment were installed at a distance of 1.5 m and 2.5 m from the smoker, perpendicular to the direction of exhalation.

**Table S1.** Number of puffs that gives the almost same amount of nicotine.

| Product | Nicotine conc. in mainstream (A) | Equivalent to one cig. (B) | Equivalent to 5.4 cigs. (C) |
| --- | --- | --- | --- |
| IQOS | 1.2 mg/stick (12puffs) | 10 puffs | 54 puffs |
| glo | 0.51 mg/ stick (12puffs) | 24 puffs | 130 puffs |
| ploom TECH | 0.25 mg/ stick (12puffs) | 49 puffs | 265 puffs |
| Mevius One | 0.97 mg/cig | 10 puffs | 54 puffs |

Nicotine concentration in mainstream aerosol/smoke from the HTP products and cigarette have been reported (A)^1-3^. The number of puffs that gives almost same amount of nicotine was calculated as follows;

The volume of using and smoking per puff was consistent with the smoker’s normal smoking behavior. Since the smoker of this test had 10 puffs with a cigarette, the number of puffs was determined to be the amount of nicotine equivalent to 10 puffs in a cigarette (B);

The average number of smoking in the room of 25 m^2^ was 5.4 cigarettes per hour^4^, so the numbers of puffs in the tests were 5.4 times of the amounts (C = 5.4 x B).

References

1. Bekki K, Inaba Y., Uchiyama S, et al. Comparison of chemicals in mainstream smoke in heat-not-burn tobacco and combustion cigarettes. *J. UOEH* **2017,** *39*, 201–207
2. Uchiyama S, Noguchi M, Takagi M, et al. Simple determination of gaseous and particulate compounds generated from heated tobacco products. *Chem. Res. Toxicol*.**2018**; *31*, 585–593
3. Endo O, Matsumoto M, Inaba Y, et.al. Nicotine, tar, and mutagenicity of mainstream smoke generated by machine smoking with International Organization for Standardization and Health Canada Intense regimes of major Japanese cigarette brands*. J Health Sci.* **2009**; *55*, 421–7.
4. Ministry of Health, Labour and Welfare. Shokuba ni okeru Judou-kitsuen-boushi-taisaku-kijun kentou-iinkai Houkokusho (The report on Committee on standards for measures to prevent second-hand smoking in the workplace) 2010 (in Japanese) <https://www.mhlw.go.jp/file/05-Shingikai-11201000-Roudoukijunkyoku-Soumuka/0000066649.pdf> (Accessed 24 September 2020)
